# The Crucial Role of the Eyes in Predicting Facial Attractiveness from Parts

**DOI:** 10.3390/bs15020141

**Published:** 2025-01-27

**Authors:** Xiaolan Gao, Hongjie Li, Xiao Han, Yong Ren, Yue Qi, Wenfeng Chen

**Affiliations:** 1 Department of Psychology, Renmin University of China, Beijing 100872, China; gaoxiaolan@ruc.edu.cn (X.G.); 2023000441@ruc.edu.cn (H.L.); hanxiao123@ruc.edu.cn (X.H.); reny18@ruc.edu.cn (Y.R.); 2 Faculty of Health and Wellness, City University of Macau, Macau, China

**Keywords:** facial attractiveness, predicting the whole face from the parts, eyes, facial parts

## Abstract

Perceiving facial attractiveness based on partial information is a common experience in social interactions, where we often view only parts of faces. However, our understanding of predictions of the whole face from its parts remains limited. This study investigated this by dividing faces into the top and bottom halves (Study 1) and the forehead, eyes, nose, and mouth (Study 2). We compared how attractiveness was predicted from these parts versus when the whole face was fully observed. In the findings, overestimations occurred when predicting from the eyes (or the top half). Similarly, predictions from the nose resulted in overestimations, though to a lesser extent. In contrast, no difference was found between the predicted and observed attractiveness of the whole face when predicting from the mouth (or the bottom half). Further analysis revealed that the eyes were the most significant predictor of facial attractiveness across all facial features. Interestingly, higher attractiveness of the eyes was associated with a greater extent of overestimations in predictions from the eyes, whereas predictions from other parts exhibited a reduced extent of overestimations. These findings implied that predictions were influenced by the eyes, underscoring the critical role the eyes play in the process of predicting facial attractiveness.

## 1. Introduction

The face is an important vehicle for social communication (Liang et al., 2010), and the evaluation of faces is a frequent occurrence in daily life. Facial attractiveness, especially, plays a significant role in various social contexts, such as trust, cooperation, friendship formation, partner selection, and job seeking (Hosoda et al., 2003; Luxen & van de Vijver, 2006; Rhodes, 2006; Shang et al., 2018, 2024; Takahash et al., 2006; Thornhill & Gangestad, 1999; Wilson & Eckel, 2006). Facial attractiveness typically refers to the attractiveness of the whole face and can be evaluated in only 100 milliseconds (Willis & Todorov, 2006). However, faces people encounter in daily scenarios are often partially visible. For instance, hats or bangs may cover the forehead, leaving the face below visible. During pandemics, masks cover the lower half of the face, revealing only the upper half. In windy or cold weather, scarves or clothing occlude faces for warmth or dust protection, leaving only the eyes exposed. Due to religious and cultural influences, women in some countries wear headscarves that cover the face, leaving only the eyes visible. In work settings, healthcare workers often wear masks or full-body protection, exposing only the eye region. Under these circumstances, the perception of facial attractiveness from partial information remains effective, yet such predictions are not necessarily accurate. Given the importance of facial attractiveness in social interactions, understanding how people predict the attractiveness of the whole face from its parts is thus a significant area of interest.

It is generally acknowledged that the key factors contributing to facial attractiveness are averageness, symmetry, and sexual dimorphism (Fink & Penton-Voak, 2002; Little et al., 2011; Rhodes, 2006). Agreement about which faces are attractive is substantial between individuals of different ages (Bronstad & Russell, 2007; Langlois et al., 2000), genders (Langlois et al., 2000), and cultures (Cunningham et al., 1995; Langlois et al., 2000). Previous studies have suggested that facial attractiveness is processed holistically (Abbas & Duchaine, 2008; Lin & Zhou, 2021; Liu & Chen, 2018; Santos & Young, 2011). It is noteworthy that numerous studies concerning facial attractiveness are performed with the full face visible. There is a relative scarcity of research that focuses on how partial facial information, such as glimpses of only certain facial regions, influences the perception of facial attractiveness.

The COVID-19 pandemic has spurred plenty of research into the effect of partial occlusion by masks on facial attractiveness. These studies have shed light on the prediction of the whole face from the visible upper half. The perceived facial attractiveness of masked and unmasked faces is considered the predicted attractiveness and observed attractiveness of the whole face, respectively (Mihara et al., 2023). If the predicted attractiveness is higher than the observed attractiveness, then the prediction results in an overestimation. Conversely, it is considered an underestimation. Nevertheless, these studies have revealed inconsistent results. Some studies have indicated that masked faces are perceived as more attractive than unmasked ones, i.e., predictions of the whole face from the top half often lead to overestimations (Hies & Lewis, 2022; Jeong et al., 2023; Kawakami et al., 2023; Lau, 2021; Patel et al., 2020). Other research has reported that masks do not affect perceived facial attractiveness, i.e., the predicted attractiveness of the whole face from the top half is comparable to the observed attractiveness of the whole face (Bennetts et al., 2022; Guo et al., 2022). In addition, some studies have shown a mix of overestimations and underestimations (Bassiri-Tehrani et al., 2022; Dudarev et al., 2022; Kamatani et al., 2021, 2023; Lee & Jeong, 2023).

The nature of occlusions or variations in methodological approaches may contribute to the observed inconsistency. For instance, masks may introduce potential confounders into prediction, such as people’s attitudes towards masks (Dudarev et al., 2022; Kamatani et al., 2021; Miyazaki & Kawahara, 2016), mask color (Prahm et al., 2023) and mask type (Hies & Lewis, 2022; Lee & Jeong, 2023), as well as other additional attributes. Alternative coverings like cards or notebooks have shown different outcomes compared to masks (Dudarev et al., 2022; Miyazaki & Kawahara, 2016). Hence, in contrast to occlusion, directly segmenting facial regions may help to yield more robust conclusions in the exploration of prediction.

While previous research has primarily focused on the top half (or masked faces), comparatively less attention has been given to the exploration of the bottom half or more detailed facial regions for prediction. One study considering the bottom half has shown contrasting prediction patterns for the bottom half versus the top half (Pazhoohi & Kingstone, 2022). Studies involving smaller facial parts have measured the attractiveness of the parts rather than the attractiveness of the whole face predicted from the parts (Liu et al., 2022; Saegusa & Watanabe, 2016) or lacked direct comparisons between the predicted and observed attractiveness of the whole face (Santos & Young, 2011). Thus, the present study sought to fill this gap by investigating the predictions of the whole face from different facial parts.

Moreover, ambiguous instructions in prior research may also lead to variability in results. When the face is partially visible with the rest occluded, participants are often asked to rate the attractiveness of the face (Dudarev et al., 2022; Hewer & Lewis, 2024; Hies & Lewis, 2022; Kamatani et al., 2021; Lau, 2021; Lee & Jeong, 2023; Oldmeadow & Koch, 2021; Patel et al., 2020; Prahm et al., 2023). However, in other studies, they are also tasked with rating the attractiveness of the image (Bassiri-Tehrani et al., 2022; Kamatani et al., 2023), the stimulus (Saegusa & Watanabe, 2016), or the facial part (Liu et al., 2022). Such instructions might result in participants rating the attractiveness of the visible part instead of the whole face it belongs to. Apart from this, there is still a possibility that researchers may conflate these two kinds of perception: perceiving the part versus perceiving the whole face that contains the part. This occurs because when the attractiveness of individual facial parts is rated higher than the whole face (Liu et al., 2022; Saegusa & Watanabe, 2016), the results are interpreted by researchers as overestimations (Liu et al., 2022; Orghian & Hidalgo, 2020). It raises a question regarding whether the attractiveness of a facial part is equivalent to the attractiveness of the whole face predicted from that part, thus suggesting a need for clarification.

Expanding upon the exploration of the patterns of predictions (i.e., the comparison between the predicted and observed attractiveness), the study delves into the efficacy of predictions from different parts. In other words, this study focuses on which facial part possesses the greatest predictive power in predicting the whole face. Numerous mask-related studies have highlighted the critical role of the eyes in predictions from the upper facial region (Lau, 2021; Oldmeadow & Koch, 2021; Pazhoohi & Kingstone, 2022; Prahm et al., 2023). Previous studies have shown that the contribution of the attractiveness of the eyes to the attractiveness of the whole face is greater compared to other facial features (Liu et al., 2022; Saegusa & Watanabe, 2016). The occlusion of the eyes, as opposed to their visibility, significantly influences perceived attractiveness (Bennetts et al., 2022; Santos & Young, 2011). Eye-tracking studies have identified the eyes (Kwart et al., 2012; Nguyen et al., 2009) or the nose (Zhang et al., 2017) as the most important region in judgments of facial attractiveness. In view of the evidence, it is reasonable to infer that the eyes are potentially the most critical predictor of whole-face attractiveness relative to other facial features.

Furthermore, the influence of individual facial parts themselves on the predictions has received scant attention in prior research. The existing body of research has demonstrated that masks may alter the perception of facial attractiveness, yet little is known about how the attractiveness of the upper facial region might modulate this effect. The current study can examine this possibility by assessing the relationship between the attractiveness of the parts and the prediction error (i.e., the difference between the predicted and observed attractiveness of the whole face) (Mihara et al., 2023). Specifically, if predictions from a specific facial part tend to overestimate whole-face attractiveness, and a positive correlation exists between this prediction error and the attractiveness of that part itself, it suggests that the higher the attractiveness of the part, the greater the degree of the overestimation. Conversely, the lack of correlation between them implies that the extent of the overestimation remains consistent regardless of the attractiveness level of the part used for prediction.

Overall, this study aimed to investigate the prediction of the attractiveness of the whole face from different parts. The faces were segmented into the top and bottom halves in Study 1 and further segmented into the forehead, eyes, nose, and mouth in Study 2, adhering to the paradigm of Liu et al. (2022) (Experiments 4A, 4B). Participants were instructed to evaluate the attractiveness of the whole face when predicted from its parts and the whole face when fully observed. The measurement of the attractiveness of the individual parts was introduced as well. We hypothesized that distinct facial parts may exhibit unique prediction patterns. Further, the eyes were hypothesized to be the most important predictor. The predictions of the whole face from the part might be influenced by the part itself. Such exploration may provide a nuanced understanding of the importance attributed to each facial part.

## 2. Study 1

The purpose of Study 1 was to explore how the attractiveness of the whole face could be predicted from its top or bottom halves and to determine further which half played a more important role in the predictive process. Participants were instructed to imagine a whole face from its segments, evaluate its attractiveness, and then evaluate the attractiveness of the whole face when fully visible. They also rated the attractiveness of each part individually. Difference analysis of the average ratings for each item was applied to discern the prediction pattern of the top or bottom half. That is, to assess whether the outcomes of prediction tend to overestimate, underestimate, or show no difference with the observed attractiveness of the whole face. Regression and correlation analyses were conducted to explore the relative importance of various facial parts in the prediction. Before the above analyses, we aimed to ascertain whether the attractiveness of the whole face predicted from its part differed from the attractiveness of the part itself by comparing the two distinct rating methods.

### 2.1. Method

#### 2.1.1. Participants

An a priori power analysis using G*Power 3.1 indicated that a minimum sample size was *N* = 28 for a medium effect size (*f* = 0.25), α = 0.05, and a power of 0.8 in a one-way, three-level repeated measures ANOVA (Faul et al., 2007). A total of 35 college students were recruited for this study (25 females, *M*_age_ = 22.65 years, *SD*_age_ = 3.03 years). Participants were all right-handed, had normal or corrected-to-normal vision, and reported no history of neurological or psychiatric diseases. The protocol was approved by the Institutional Review Board of the Department of Psychology, Remin University of China. All participants provided informed consent before participating and were provided with debriefing and compensation upon completion.

#### 2.1.2. Design

This study used a within-participant design. The independent variable was the visible region (top half, bottom half, whole face). The dependent variable was the attractiveness ratings of the imagined whole face from the two halves (predicted attractiveness of the whole face) and the attractiveness ratings of the whole face (observed attractiveness of the whole face).

#### 2.1.3. Materials

**Figure 1:**
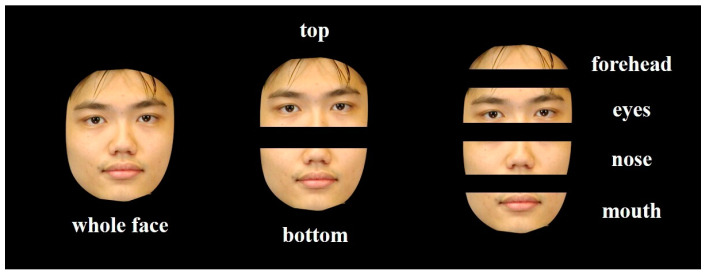
Examples of face stimuli used in Study 1 and Study 2.

The face stimuli were identical to those used in Experiment 4A of Liu et al. (2022), as shown in Figure 1. The stimuli were selected from the CUHK Face Sketch Database (CUFS, http://mmlab.ie.cuhk.edu.hk/datasets.html (accessed on 19 July 2023)) (Wang & Tang, 2009). The database includes 188 faces from the Chinese University of Hong Kong (CUHK) student data set, with 134 male faces. In total, 90 male faces were chosen and further cropped to remove hair and external features against a black background. Each face image was then segmented into the top half and the bottom half. With the addition of the whole face, there was a collection of 270 images (90 × 3).

#### 2.1.4. Procedure

**Figure 2:**
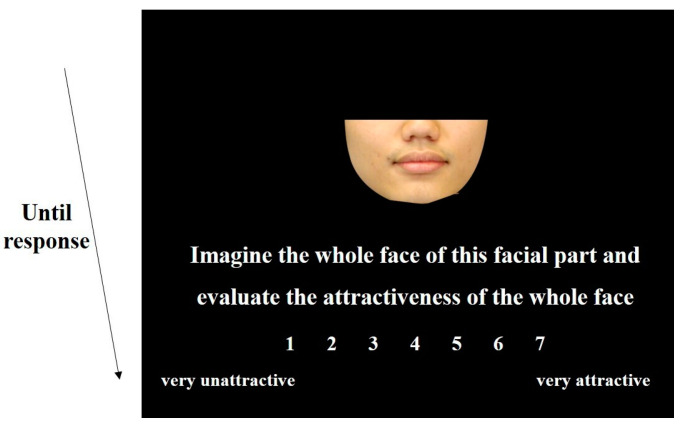
Example trial of Study 1 and Study 2.

As shown in Figure 2, a face stimulus was presented in the center of the screen in each trial, with instructions and a rating scale ranging from 1 (very unattractive) to 7 (very attractive) presented at the bottom. The study consisted of three experiments. The first experiment consisted of two blocks. The top half or bottom half of a face identity was randomly assigned to one of these two blocks, such that different parts of the same face identity would not appear in one block. Participants were instructed to imagine the whole face from the visible part and evaluate the attractiveness of the imagined whole face. In the second experiment, the whole face was presented, and participants were instructed to rate the attractiveness of the whole face. In the third experiment, the stimuli of the two blocks of the first experiment were represented. This time, participants were required to evaluate the attractiveness of the facial parts. The study consisted of 5 blocks, with 450 trials in total. The presentation order of the 90 face identities in the first block was randomized for each participant. The order of face identities in the following blocks corresponded to that of the first block, which was consistent with the previous research (Liu et al., 2022). Blocks were separated by a 1 min rest break. Participants had no time limit when clicking on the values to rate in each trial, with an average response time of approximately 2 s. This study lasted about 20 min.

### 2.2. Results

#### 2.2.1. Descriptive Statistics

The average attractiveness ratings of each item of this study are shown in Table 1, and the correlation analysis is shown in Table 2. To validate the reliability of the attractiveness ratings, the inter-rater agreement was calculated among participants using Cronbach’s alpha coefficient and the intraclass correlation coefficient (ICC) (Liu et al., 2022; Santos & Young, 2011). This step was important given that while previous studies have demonstrated good inter-rater agreement for the attractiveness ratings of the whole face (Todorov et al., 2015) and the facial parts (Liu et al., 2022), the reliability of attractiveness ratings of the imagined whole face from partial information had yet to be established. The results revealed high reliability of the attractiveness ratings, as shown in Table 1.

#### 2.2.2. Difference Between the Predicted Attractiveness of the Whole Face from the Parts and the Attractiveness of the Parts

All analyses below applied the Greenhouse–Geisser correction when the sphericity assumption was violated and the Bonferroni correction for the post hoc tests.

We first conducted a two-way repeated measures ANOVA with the visible region (top half, bottom half) and the rating method (rating the whole face imagined from the parts, rating the parts) as the independent variables. The results showed that the main effect of the visible region was significant (*F*(1, 89) = 116.61, *p* < 0.001, $\eta_{p}^{2}$ = 0.57). The main effect of the rating method was significant (*F*(1, 89) = 435.65, *p* < 0.001, $\eta_{p}^{2}$ = 0.83). The interaction between the two factors was significant (*F*(1, 89) = 39.23, *p* < 0.001, $\eta_{p}^{2}$ = 0.31). Simple effects tests showed that the predicted attractiveness from the top was significantly higher than the attractiveness of the top (*F*(1, 89) = 355.93, *p* < 0.001, $\eta_{p}^{2}$ = 0.80). The predicted attractiveness from the bottom was also significantly higher than the attractiveness of the bottom (*F*(1, 89) = 126.48, *p* < 0.001, $\eta_{p}^{2}$ = 0.59). The interaction arose due to the more pronounced difference between the predicted attractiveness of the whole face and the attractiveness of the top.

Therefore, the results revealed a significant difference between the attractiveness of a facial part and the attractiveness of the whole face as perceived from that part.

#### 2.2.3. Difference Between the Predicted and Observed Attractiveness of the Whole Face

**Figure 3:**
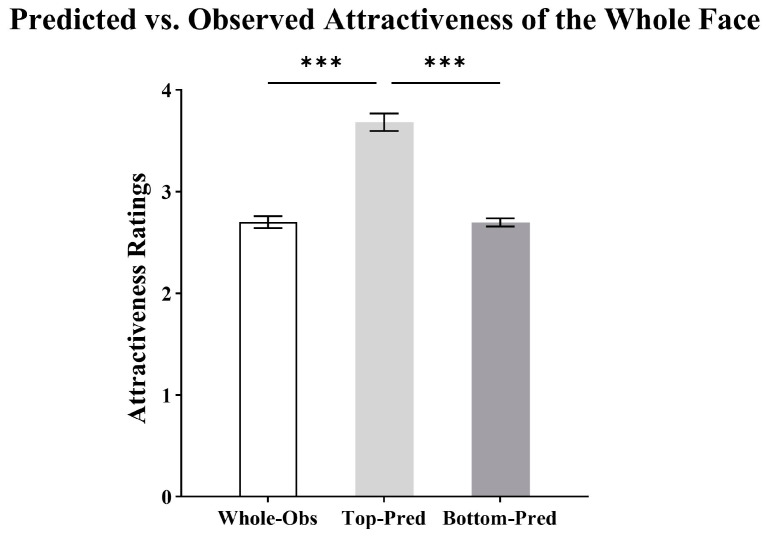
Attractiveness ratings (means and SEM) for the predicted attractiveness of the whole face from the top half and the bottom half, as well as the observed attractiveness of the whole face (*** *p* < 0.001).

As shown in Figure 3, the independent variable was the visible region (whole face, top half, bottom half), and the dependent variable was the predicted or observed attractiveness of the whole face. A one-way, three-level repeated measures ANOVA showed that the main effect of the visible region was significant (*F*(1.31, 116.64) = 137.54, *p* < 0.001, $\eta_{p}^{2}$ = 0.61).

Pairwise comparisons revealed that the predicted attractiveness from the top was significantly higher than the observed attractiveness of the whole face (*M_diff_* = 0.98, *SD* = 0.06, *p* < 0.001). There was no significant difference between the predicted attractiveness from the bottom and the observed attractiveness of the whole face (*M_diff_* = −0.003, *SD* = 0.05, *p* > 0.999). In addition, the predicted attractiveness from the top was significantly higher than that from the bottom (*M_diff_* = 0.99, *SD* = 0.09, *p* < 0.001).

#### 2.2.4. Importance of the Parts in Predicting the Whole Face from the Parts

##### Regression Analysis of the Predictions

To explore which part was more important in predicting the whole face, we conducted regression analysis with the observed attractiveness of the whole face as the predicted variable and the predicted attractiveness of the whole face from the parts as the predictors. The results showed that either the top or the bottom alone significantly predicted whole-face attractiveness (Top: *F*(1, 88) = 100.46, *p* < 0.001, adjusted *R*^2^ = 0.53. Bottom: *F*(1, 88) = 29.93, *p* < 0.001, adjusted *R*^2^ = 0.25). When combined, both the two halves significantly predicted whole-face attractiveness (*F*(2, 87) = 107.46, *p* < 0.001, adjusted *R*^2^ = 0.71), as shown in Table 3.

As evidenced by higher *R*^2^ in the separate prediction models and higher *β* in the combined prediction model, the top was a more effective predictor of whole-face attractiveness than the bottom.

##### Correlation Analysis of the Prediction Error with the Attractiveness of the Parts

To examine whether the prediction of the whole face from the part would be influenced by the part itself, correlation analysis was conducted between the prediction error and the attractiveness of the part itself as well as other parts.

As shown in Table 4, the results showed that the prediction error when predicting from the top significantly and positively correlated with the attractiveness of the top itself, with a weak correlation to that of the bottom. In contrast, the prediction error when predicting from the bottom did not significantly correlate with the attractiveness of the bottom itself. It significantly and negatively correlated with the attractiveness of the top.

**Figure 4:**
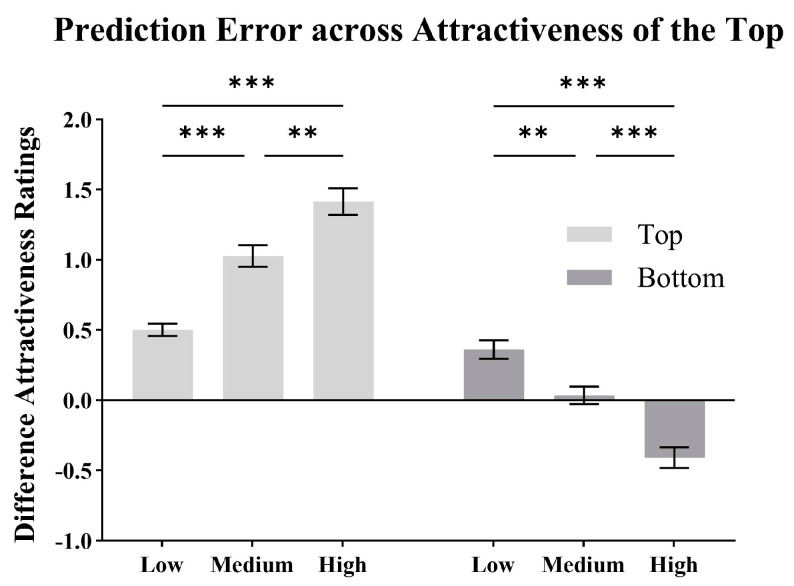
Difference attractiveness ratings (means and SEM) for the prediction error (i.e., predicted attractiveness minus observed attractiveness) when predicting from the top half and the bottom half, with the faces categorized based on the attractiveness of the top half (** *p* < 0.01; *** *p* < 0.001).

To better illustrate the influence of the top on the predictions of the whole face from both the top and bottom, we categorized the faces into three groups based on the attractiveness ratings of the top. We analyzed the prediction error across different levels of attractiveness of the top, as shown in Figure 4.

The ratings of the top among the three groups: low (*M* = 2.56, *SD* = 0.37), medium (*M* = 3.35, *SD* = 0.26), and high (*M* = 4.21, *SD* = 0.35) showed a significant difference (*F*(2, 87) = 188.21, *p* < 0.001, $\eta_{p}^{2}$ = 0.82; *N* = 30 for each group). The independent variables were the visible region (top half, bottom half) and the attractiveness group of the top (low, medium, high). The dependent variable was the prediction error (i.e., predicted attractiveness minus observed attractiveness). A two-way mixed-design ANOVA showed that the main effect of the visible region was significant (*F*(1, 87) = 345.47, *p* < 0.001, $\eta_{p}^{2}$ = 0.80). The main effect of the attractiveness group of the top was not significant (*F*(2, 87) = 0.87, *p* = 0.42, $\eta_{p}^{2}$ = 0.02). The interaction between the two factors was significant (*F*(2, 87) = 84.16, *p* < 0.001, $\eta_{p}^{2}$ = 0.66). Simple effects analyses were as follows.

For the top, the effect of the attractiveness group was significant (*F*(2, 87) = 37.21, *p* < 0.001, $\eta_{p}^{2}$ = 0.46). Pairwise comparisons showed that the prediction error of the high group was significantly higher than that of the medium (*M_diff_* = 0.39, *SD* = 0.11, *p* = 0.001) and the low group (*M_diff_* = 0.91, *SD* = 0.11, *p* < 0.001). The medium group was significantly higher than the low group (*M_diff_* = 0.53, *SD* = 0.11, *p* < 0.001).

For the bottom, the simple effect of the attractiveness group was significant (*F*(2, 87) = 33.18, *p* < 0.001, $\eta_{p}^{2}$ = 0.43). Pairwise comparisons showed that the prediction error of the high group was significantly lower than that of the medium (*M_diff_* = −0.44, *SD* = 0.10, *p* < 0.001) and the low group (*M_diff_* = −0.77, *SD* = 0.10, *p* < 0.001). The medium group was significantly lower than the low group (*M_diff_* = −0.33, *SD* = 0.10, *p* = 0.003).

### 2.3. Discussion

The results of Study 1 showed that the predicted attractiveness of the whole face from the top half was significantly higher than the observed attractiveness of the whole face. The predicted attractiveness from the bottom half had no significant difference from the observed attractiveness. In other words, predictions from the top half tended to overestimate whole-face attractiveness, while those from the bottom half were comparable to it. Before this, we contrasted the two rating methods when the individual parts were presented. It was demonstrated that the attractiveness of the whole face as perceived from the part was different from the attractiveness of that part. This finding emphasized the necessity of distinguishing between these two kinds of perception to prevent potential confusion.

The results further indicated that the top half held greater importance than the bottom half in predicting the whole face from its parts. On the one hand, the top half provided a better prediction of whole-face attractiveness, as suggested in the regression analysis. On the other hand, the attractiveness of the top influenced not only predictions from the top itself but also predictions from the bottom. Surprisingly, predictions from the bottom were not influenced by the bottom itself because there was no significant correlation between the prediction error and its attractiveness. Specifically, the higher the attractiveness of the top, the greater the extent of overestimations in predictions from the top. When predicting from the bottom, a higher attractiveness of the top was associated with underestimations, whereas a lower attractiveness of the top resulted in overestimations.

It appears that the top and bottom halves of the face differ in terms of prediction patterns and relative importance. However, it is uncertain whether the disparity arises from the distinct minor facial features they each contain, such as the forehead or the eyes in the top half, or the mouth in the bottom half.

## 3. Study 2

Study 2 aimed to replicate and extend the findings of Study 1 by delving deeper into predictions from even smaller facial parts. We placed a particular focus on the eyes and hypothesized that the eyes would hold greater predictive power compared to other facial parts. Drawing on previous studies that have highlighted the significance of the eyes in predicting facial attractiveness from the upper facial region (Lau, 2021; Oldmeadow & Koch, 2021; Pazhoohi & Kingstone, 2022; Prahm et al., 2023), we hypothesized that the eyes would exhibit patterns akin to those of the top half and play a leading role in the predictions from various parts.

### 3.1. Method

#### 3.1.1. Participants

An a priori power analysis using G*Power 3.1 indicated that the required minimum sample size was *N* = 21, with the parameters of medium effect size (*f* = 0.25), α = 0.05, and power of 0.8 in a one-way, five-level repeated measures ANOVA (Faul et al., 2007). A total of 38 participants were recruited for this study (20 females, *M*_age_ = 21.03 years, *SD*_age_ = 1.60 years). All participants were right-handed, had normal or corrected-to-normal vision, and did not participate in Study 1.

#### 3.1.2. Design

This study used a within-participant design. The independent variable was the visible region (forehead, eyes, nose, mouth, whole face). The dependent variable was the attractiveness ratings of the imagined whole face from the four parts and the attractiveness ratings of the whole face.

#### 3.1.3. Materials

The face stimuli were identical to those used in Study 1 and Experiment 4B by Liu et al. (2022), as shown in Figure 1. The 90 male faces were divided into the forehead, eyes, nose, and mouth. Including the whole face, this resulted in a total of 450 images (90 × 5).

#### 3.1.4. Procedure

The experimental procedure was similar to Study 1, as shown in Figure 2. The study also consisted of three experiments. The first experiment consisted of four blocks. Four parts of a face identity (forehead, eyes, nose, or mouth) were randomly assigned to one of these four blocks without repetition within the same block. Participants were asked to imagine the whole face from the presented part and judge the attractiveness of the imagined whole face. The second experiment also consisted of four blocks. Four parts of a face identity were again randomly assigned to one of these four blocks. Participants were instructed to rate the attractiveness of the facial parts. These two experiments were randomly presented to each participant. In the third experiment, the whole face was presented, and participants were instructed to rate the attractiveness of the whole face. The study consisted of 9 blocks and 810 trials (9 × 90). In each block, the sequence of the 90 facial identities was randomized. Blocks were separated by a 1 min rest break. The average duration of this study was approximately 45 min.

### 3.2. Results

#### 3.2.1. Descriptive Statistics

The average attractiveness ratings and inter-rater agreement of each item of this study are shown in Table 5, and the correlation analysis is shown in Table 6.

#### 3.2.2. Difference Between the Predicted Attractiveness of the Whole Face from the Parts and the Attractiveness of the Parts

We first examined whether there was a difference between the predicted attractiveness of the whole face from the parts and the attractiveness of that part. A two-way repeated measures ANOVA was conducted with the visible region (forehead, eyes, nose, mouth) and the rating method (rating the whole face imagined from the parts, rating the parts) as the independent variables. The main effect of the visible region was significant (*F*(2.23, 201.64) = 42.43, *p* < 0.001, $\eta_{p}^{2}$ = 0.32). The main effect of the rating method was significant (*F*(1, 89) = 86.57, *p* < 0.001, $\eta_{p}^{2}$ = 0.49), suggesting that the predicted attractiveness from the parts was significantly higher than the attractiveness of these respective parts. The interaction between the two factors was not significant (*F*(2.92, 260.09) = 1.95, *p* = 0.123, $\eta_{p}^{2}$ = 0.02).

Thus, even considering the smaller facial regions, the attractiveness of the whole face predicted from the parts was generally higher than the attractiveness of those parts.

#### 3.2.3. Difference Between the Predicted and Observed Attractiveness of the Whole Face

**Figure 5:**
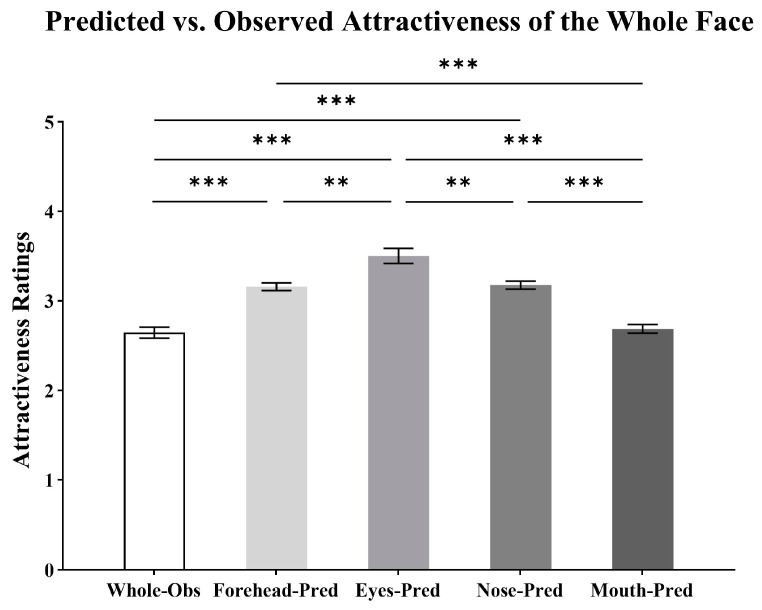
Attractiveness ratings (means and SEM) for the predicted attractiveness of the whole face from the forehead, eyes, nose, and mouth, as well as the observed attractiveness of the whole face (** *p* < 0.01; *** *p* < 0.001).

As shown in Figure 5, the independent variable was the visible region (whole face, forehead, eyes, nose, mouth), and the dependent variable was the predicted or observed attractiveness of the whole face. A one-way, five-level repeated measures ANOVA indicated a significant main effect of visible region (*F*(2.61, 231.82) = 51.59, *p* < 0.001, $\eta_{p}^{2}$ = 0.37).

Pairwise comparisons showed that the predicted attractiveness from the forehead (*M_diff_* = 0.51, *SD* = 0.07, *p* < 0.001), eyes (*M_diff_* = 0.86, *SD* = 0.06, *p* < 0.001), or nose (*M_diff_* = 0.53, *SD* = 0.06, *p* < 0.001) was significantly higher than the observed attractiveness of the whole face. There was no significant difference between the predicted attractiveness from the mouth and the observed attractiveness of the whole face (*M_diff_* = 0.04, *SD* = 0.06, *p* > 0.999). In addition, the predicted attractiveness from the eyes was significantly higher than that from the forehead (*M_diff_* = 0.34, *SD* = 0.09, *p* = 0.002), nose (*M_diff_* = 0.33, *SD* = 0.09, *p* = 0.004), and mouth (*M_diff_* = 0.81, *SD* = 0.10, *p* < 0.001). The nose was significantly higher than the mouth (*M_diff_* = 0.49, *SD* = 0.06, *p* < 0.001) and comparable to the forehead (*M_diff_* = 0.02, *SD* = 0.06, *p* > 0.999). The mouth was significantly lower than the forehead (*M_diff_* = −0.47, *SD* = 0.06, *p* < 0.001).

#### 3.2.4. Importance of the Parts in Predicting the Whole Face from the Parts

##### Regression Analysis of the Predictions

Regression analyses were conducted with the observed attractiveness of the whole face as the predicted variable and the predicted attractiveness of the whole face from the parts as the predictors. The results showed that the eyes, nose, or mouth alone significantly predicted whole-face attractiveness (Eyes: *F*(1, 88) = 109.90, *p* < 0.001, adjusted *R*^2^ = 0.55. Nose: *F*(1, 88) = 23.20, *p* < 0.001, adjusted *R*^2^ = 0.20. Mouth: *F*(1, 88) = 13.59, *p* < 0.001, adjusted *R*^2^ = 0.12). However, the forehead did not significantly predict it (*F*(1, 88) = 1.03, *p* = 0.34, adjusted *R*^2^ < 0.001). When combined, the eyes, nose, and mouth significantly predicted whole-face attractiveness, except for the forehead (*F*(4, 85) = 56.10, *p* < 0.001, adjusted *R*^2^ = 0.71), as shown in Table 7.

As indicated by the highest *R*^2^ in the separate prediction models and the highest *β* in the combined prediction model, the eyes were the most effective predictor of whole-face attractiveness relative to other parts.

##### Correlation Analysis of the Prediction Error with the Attractiveness of the Parts

As shown in Table 8, correlation analysis showed that the prediction error when predicting from the eyes significantly and positively correlated with the attractiveness of the eyes themselves, with a weak correlation to that of the mouth. Surprisingly, the prediction error when predicting from the forehead, nose, and mouth all significantly and negatively correlated with the attractiveness of the eyes, with weak or non-significant correlation to the attractiveness of the part itself or other parts. Although correlations related to the prediction error from the forehead were present, it was reasonable not to consider them since the forehead could not predict whole-face attractiveness.

**Figure 6:**
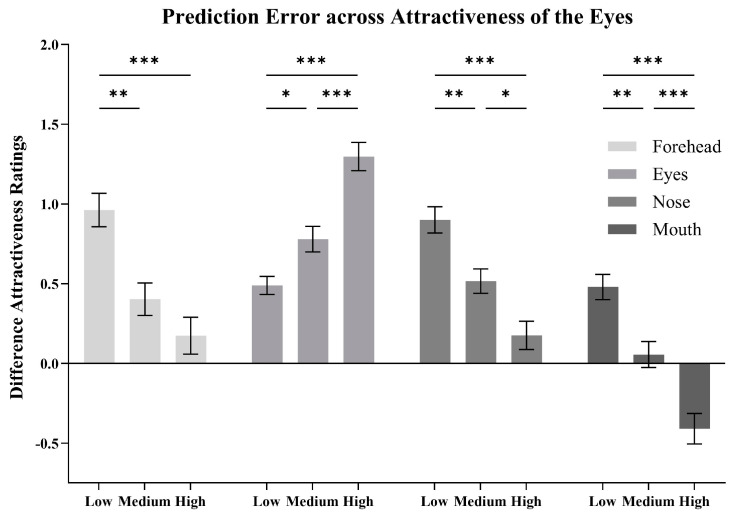
Difference attractiveness ratings (means and SEM) for the prediction error (i.e., predicted attractiveness minus observed attractiveness) when predicting from the forehead, eyes, nose, and mouth, with the faces categorized based on the attractiveness of the eyes (* *p* < 0.05; ** *p* < 0.01; *** *p* < 0.001).

To better illustrate the impact of the eyes on the predictions of the whole face from all four parts, we categorized the faces into three groups based on the attractiveness ratings of the eyes and analyzed the prediction error across different levels of attractiveness of the eyes, as shown in Figure 6.

The ratings of the eyes among the three groups: low (*M* = 2.62, *SD* = 0.43), medium (*M* = 3.41, *SD* = 0.15), and high (*M* = 4.31, *SD* = 0.36) showed a significant difference (*F*(2, 87) = 186.58, *p* < 0.001, $\eta_{p}^{2}$ = 0.81; *N* = 30 for each group). The independent variables were the visible region (eyes, nose, mouth) and the attractiveness group of the eyes (low, medium, high). The dependent variable was the prediction error. A two-way mixed-design ANOVA indicated significant main effects of the visible region (*F*(3, 261) = 63.46, *p* < 0.001, $\eta_{p}^{2}$ = 0.42) and attractiveness group of the eyes (*F*(2, 87) = 10.43, *p* < 0.001, $\eta_{p}^{2}$ = 0.19). The interaction between the two factors was also significant (*F*(6, 261) = 31.18, *p* < 0.001, $\eta_{p}^{2}$ = 0.42). Simple effects analyses were then conducted.

For the forehead, the effect of the attractiveness group was significant (*F*(2, 87) = 14.10, *p* < 0.001, $\eta_{p}^{2}$ = 0.25). Pairwise comparisons showed that the prediction error of the high group was significantly lower than that of the low group (*M_diff_* = −0.79, *SD* = 0.15, *p* < 0.001) and compared to that of the medium group (*M_diff_* = −0.23, *SD* = 0.15, *p* = 0.41). The medium group was significantly lower than the low group (*M_diff_* = −0.56, *SD* = 0.15, *p* = 0.001).

For the eyes, the simple effect of the attractiveness group was significant (*F*(2, 87) = 28.70, *p* < 0.001, $\eta_{p}^{2}$ = 0.40). Pairwise comparisons showed that the prediction error of the high group was significantly higher than that of the medium (*M_diff_* = 0.52, *SD* = 0.11, *p* < 0.001) and the low group (*M_diff_* = 0.81, *SD* = 0.11, *p* < 0.001). The medium group was significantly higher than the low group (*M_diff_* = 0.29, *SD* = 0.11, *p* = 0.026).

For the nose, the simple effect of the attractiveness group was significant (*F*(2, 87) = 19.06, *p* < 0.001, $\eta_{p}^{2}$ = 0.31). Pairwise comparisons showed that the prediction error of the high group was significantly lower than that of the medium (*M_diff_* = −0.34, *SD* = 0.12, *p* = 0.014) and the low group (*M_diff_* = −0.73, *SD* = 0.12, *p* < 0.001). The medium group was significantly lower than the low group (*M_diff_* = −0.38, *SD* = 0.142 *p* = 0.005).

For the mouth, the simple effect of the attractiveness group was significant (*F*(2, 87) = 26.91, *p* < 0.001, $\eta_{p}^{2}$ = 0.38). Pairwise comparisons showed that the prediction error of the high group was significantly lower than that of the medium (*M_diff_* = −0.47, *SD* = 0.12, *p* < 0.001) and the low group (*M_diff_* = −0.89, *SD* = 0.12, *p* < 0.001). The medium group was significantly lower than the low group (*M_diff_* = −0.42, *SD* = 0.12, *p* = 0.002).

### 3.3. Discussion

Study 2 confirmed the main findings from Study 1 with additional insights. The results showed that predictions from the eyes tended to overestimate whole-face attractiveness. Predictions from the nose also showed a tendency to overestimate whole-face attractiveness to a lesser degree, while those from the mouth closely approximated it. Despite the overestimation observed in predictions from the forehead, regression analyses revealed that the forehead was not a predictor of whole-face attractiveness. This may be attributed to its relatively smaller size or lack of features, which may not provide adequate information for forming a representation of the whole face. The results further suggested a trend wherein predictions focusing on regions higher on the face were more likely to yield overestimations, consistent with Study 1. The overestimation from the eyes was the most substantial among all facial features.

Critically, the results showed that the eyes were of the greatest significance in predicting the whole face from its parts. On the one hand, the eyes had a stronger predictive power compared to other features. On the other hand, predictions of the whole face from its parts were predominantly influenced by the eyes, regardless of the selected part for prediction. When the eyes were more attractive, the extent to which individuals overestimated whole-face attractiveness was greater when predicting from the eyes. Conversely, such overestimations were diminished when predictions were made from the other parts, such as the nose and mouth, and even shifted to underestimations when predictions were made from the mouth.

## 4. General Discussion

The current study explored the prediction of facial attractiveness from the facial parts. The face was divided into the top and bottom halves in Study 1 and the forehead, eyes, nose, and mouth in Study 2. We compared the attractiveness of the whole face predicted from different facial parts and the attractiveness of the whole face when it was fully observed. We also explored the importance of each part in the prediction. The results revealed that participants overestimated facial attractiveness when predicting from the top half (Study 1), eyes, or nose (Study 2). The predicted attractiveness from the bottom half (Study 1) or mouth (Study 2) was comparable to the observed attractiveness of the whole face. Furthermore, the eyes (the top) were the most important predictors compared to the forehead, nose, and mouth (the bottom). The prediction error when predicting from the eyes (the top) correlated with the attractiveness of the eyes (the top) themselves. Moreover, the prediction error when predicting from other parts also correlated with the attractiveness of the eyes (the top), with weak or non-significant correlation to that of the part itself or other parts. Specifically, the higher the attractiveness of the eyes (the top), the greater the extent of overestimations in predictions from the eyes (the top). In contrast, an increased attractiveness of the eyes (the top) was linked to a decrease in the extent of overestimations when predictions were made from the nose, forehead, and mouth (the bottom).

Overestimations occurred when predicting from the top half, eyes, or nose, while the bottom half or mouth yielded comparable predictions. The overestimation from the top half aligns with studies indicating that masking can enhance perceived facial attractiveness (Hies & Lewis, 2022; Jeong et al., 2023; Kawakami et al., 2023; Lau, 2021; Patel et al., 2020). It is noteworthy that the study addresses potential confounds introduced by masks by excluding them and clarifies ambiguities that may arise from the instructions by comparing the two kinds of attractiveness ratings, thereby providing a purer predictive process. Moreover, the prediction pattern of the bottom half differed from that of the top half, which is consistent with previous research (Pazhoohi & Kingstone, 2022).

Collectively, Study 1 and Study 2 revealed that the prediction patterns varied with the facial parts considered. Previous studies have suggested that the overestimation can be attributed to the internal representation of a prototypical face (Kramer & Jones, 2022; Liu et al., 2022; Orghian & Hidalgo, 2020). That is, individuals use a prototypical face to fill in missing information in incomplete faces (Orghian & Hidalgo, 2020), with the prototypical face (average or typical faces) being perceived as more attractive (Langlois & Roggman, 1990; Rhodes et al., 1999). However, the results of the present study cannot be fully explained by the internal representation mechanisms, particularly for the bottom half and the mouth. Thus, the distinct prediction patterns underscore the need to consider the uniqueness of each facial region.

Moreover, predictions from the eyes closely resembled those from the top half. Despite the top half also comprising the forehead, the analysis established that the forehead did not significantly contribute to predicting whole-face attractiveness. Consequently, it is logical to deduce that the predictions from the top half are primarily driven by the eyes. This is in line with the previous literature that has highlighted the significance of the eyes in predicting facial attractiveness when the top half is visible (Lau, 2021; Oldmeadow & Koch, 2021; Pazhoohi & Kingstone, 2022; Prahm et al., 2023).

Further analysis revealed that the eyes emerged as the most important predictor of whole-face attractiveness. Previous research has suggested that the attractiveness of the eyes contributes most significantly to the attractiveness of the whole face (Liu et al., 2022; Saegusa & Watanabe, 2016). The findings of this study have discrepancies from prior research while also demonstrating a complementary relationship. This study reveals that when faces are partially visible, the eyes have the greatest predictive efficacy to predict whole-face attractiveness compared to other facial features.

Furthermore, the attractiveness of the eyes positively correlated with the prediction error of the eyes and negatively correlated with that of other parts. That is, higher attractiveness of the eyes led to a greater extent of overestimations in predictions of the whole face from the eyes while reducing the extent of overestimations in predictions from the nose and increasing the likelihood of underestimations in predictions from the mouth. However, this effect was not as pronounced for other facial parts. These findings suggest that the degree of prediction is not consistent but varies across different levels of attractiveness of the eyes, even when other parts are used for prediction. Thus, the eyes have a prominent influence on these predictions.

In conclusion, the eyes play a decisive role in the prediction of facial attractiveness from all the parts. Additionally, their predictive power surpassed other facial features. These findings contribute to extensive prior research that has emphasized the pivotal role of the eyes in facial attractiveness perception. In these studies, the eyes are consistently identified as crucial for judging attractiveness, irrespective of whether faces are fully visible (Diego-Mas et al., 2020; Kwart et al., 2012; Nguyen et al., 2009; Paunonen et al., 1999) or partially obscured (Lau, 2021; Oldmeadow & Koch, 2021; Pazhoohi & Kingstone, 2022; Prahm et al., 2023; Santos & Young, 2011). Their significance is not limited to attractiveness but extends to multiple facets, such as face identification, expression categorization, gender recognition, race perception, and so on (Dupuis-Roy et al., 2009; Lewis & Edmonds, 2003; Royer et al., 2018; Schyns et al., 2007; Yong et al., 2020).

Notably, the uniqueness of the eyes involved in the predictive process aligns with prior research on the eye anchoring mechanism (Nemrodov & Itier, 2011) and the eye detector mechanism (Bentin et al., 1996). The research by Nemrodov et al. (2014) has further highlighted the sensitivity and distinctiveness of the eyes in the holistic processing of faces. The researchers have proposed that the eyes act as an anchor point or reference point from which the position and orientation of other features are coded to generate a face percept (Nemrodov & Itier, 2011). The eye detector is specialized in detecting eyes to anchor the face percept at the eyes (Nemrodov et al., 2014). Considering the empirical evidence indicating that facial attractiveness relies on a holistic representation (Abbas & Duchaine, 2008; Lin & Zhou, 2021; Liu & Chen, 2018; Santos & Young, 2011), the current study extends prior conclusions by suggesting that the eyes play a central role in the holistic processing of facial attractiveness.

The current work expands the scope beyond the upper facial region that previous research mainly focused on and concurrently compares the prediction patterns across various facial parts. It provides a full picture of predictions and deepens our understanding of the relationship between the part and the whole, thus making important theoretical contributions to the existing literature. From the practical aspect, the study suggests an alternative approach that diverges from the traditional approach of beautifying specific facial regions. Recognizing the prominent role of the eyes in predicting facial attractiveness, appropriately obscuring non-ocular areas may be an effective strategy to enhance one’s facial appearance.

## 5. Limitations and Future Directions

The current study has several limitations warranting future research. First, the terms ‘eyes,’ ‘nose,’ and ‘mouth’ refer to the features as well as the broader regions surrounding them rather than solely the features. Future research might benefit from refinements of these regions to isolate the effects of the specific features. Second, the sample of this study was limited to young individuals, and the face images were of Asian males. Future studies should investigate whether the gender of faces and the gender of participants affect the stability of the results. To enhance the generalizability of the conclusions, it would be beneficial to include samples and face stimuli with diverse demographic characteristics and to conduct cross-cultural research. Third, future research could enhance the robustness of the findings by conducting multiple measurements of the same stimuli. Fourth, the study focused on the facial regions without addressing scenarios where parts of a complete face are blurred or missing (e.g., random pixel elimination in images, Orghian & Hidalgo, 2020). This is because faces with missing parts retain a high degree of integrity, and further requesting the imagination of a whole face may result in a lack of distinction between the imagined and original faces. Fifth, while this study has provided empirical evidence about attractiveness, future research could extend this exploration to other facial trait inferences, such as trustworthiness and dominance, to enrich our understanding of how facial parts contribute to other social perceptions. Sixth, from a neuropsychological perspective, follow-up research could use electrophysiological methods to investigate the brain process involved in predicting facial attractiveness from its parts. This approach may yield more insights into the neural mechanisms underlying attractiveness prediction.

## 6. Conclusions

The study explored the prediction of the whole face from its parts with fine segmentation of the faces. It revealed that predictions from the eyes yielded the highest attractiveness scores, thus resulting in the greatest overestimations. The predictive power of the eyes also surpassed that of other facial features. The eyes were also the key determinant in predictions from the top half. Moreover, the attractiveness of the eyes could disproportionately sway the magnitude of the predictions, even when other parts were under consideration. Specifically, the extent of overestimations increased with the increase in attractiveness of the eyes when predicting from the eyes, while it decreased when predicting from other facial parts. Therefore, the eyes, as a key facial feature, play a critical role in the predictive process, leading to any prediction of the whole face from its parts inevitably influenced by the eyes.

## Figures and Tables

**Table 1:** Attractiveness ratings and their reliability in Study 1 (*N*_item_ = 90).

	Mean	SD	Cronbach’s α	ICC
Predicted attractiveness from the top	3.68	0.81	0.96	0.94
Predicted attractiveness from the bottom	2.70	0.37	0.88	0.76
Attractiveness of the top	3.37	0.75	0.96	0.93
Attractiveness of the bottom	2.53	0.32	0.87	0.73
Observed attractiveness of the whole face	2.70	0.55	0.93	0.88

**Table 2:** Correlations between the predicted attractiveness, the observed attractiveness, and the attractiveness of the parts in Study 1 (*N*_item_ = 90).

	1	2	3	4	5
1 Predicted attractiveness from the top	1				
2 Predicted attractiveness from the bottom	0.11	1			
3 Attractiveness of the top	0.98 ***	0.09	1		
4 Attractiveness of the bottom	0.18	0.92 ***	0.16	1	
5 Observed attractiveness of the whole face	0.73 ***	0.50 ***	0.74 ***	0.53 ***	1

Note: *** *p* < 0.001.

**Table 3:** Multiple regression analysis for predicting the observed attractiveness of the whole face based on the predicted attractiveness from the parts in Study 1.

	*B*	*SE*	*β*	*t*	*p*
Predicted attractiveness from the top	0.46	0.04	0.68	11.76	<0.001
Predicted attractiveness from the bottom	0.62	0.09	0.43	7.35	<0.001

**Table 4:** Correlations between the prediction error when predicting from the parts and the attractiveness of the parts in Study 1.

	Top	Bottom
Predicted error from the top	0.70 ***	−0.26 *
Predicted error from the bottom	−0.77 ***	−0.12

Note: * *p* < 0.05; *** *p* < 0.001.

**Table 5:** Attractiveness ratings and their reliability in Study 2 (*N*_item_ = 90).

	Mean	SD	Cronbach’s α	ICC
Predicted attractiveness from the forehead	3.16	0.41	0.87	0.72
Predicted attractiveness from the eyes	3.50	0.80	0.95	0.93
Predicted attractiveness from the nose	3.18	0.43	0.86	0.78
Predicted attractiveness from the mouth	2.69	0.46	0.90	0.82
Attractiveness of the forehead	3.05	0.39	0.87	0.70
Attractiveness of the eyes	3.44	0.77	0.95	0.92
Attractiveness of the nose	3.05	0.44	0.87	0.81
Attractiveness of the mouth	2.58	0.46	0.90	0.84
Observed attractiveness of the whole face	2.65	0.58	0.92	0.89

**Table 6:** Correlations between the predicted attractiveness, the observed attractiveness, and the attractiveness of the parts in Study 2 (*N*_item_ = 90).

	1	2	3	4	5	6	7	8
1 Predicted attractiveness from the forehead	1							
2 Predicted attractiveness from the eyes	0.14	1						
3 Predicted attractiveness from the nose	−0.06	0.20	1					
4 Predicted attractiveness from the mouth	0.14	0.05	0.22 *	1				
5 Attractiveness of the forehead	0.88 ***	0.11	−0.11	0.16	1			
6 Attractiveness of the eyes	0.13	0.97 ***	0.16	0.01	0.12	1		
7 Attractiveness of the nose	0.11	0.25 *	0.87 ***	0.21 *	0.09	0.22 *	1	
8 Attractiveness of the mouth	0.14	0.11	0.24 *	0.93 ***	0.15	0.06	0.22 *	1
9 Observed attractiveness of the whole face	0.11	0.75 ***	0.46 ***	0.37 ***	0.09	0.70 ***	0.45 ***	0.44 ***

Note: * *p* < 0.05; *** *p* < 0.001.

**Table 7:** Multiple regression analysis for predicting the observed attractiveness of the whole face based on the predicted attractiveness from the parts in Study 2.

	*B*	*SE*	*β*	*t*	*p*
Predicted attractiveness from the forehead	−0.02	0.08	−0.01	−0.20	0.84
Predicted attractiveness from the eyes	0.49	0.04	0.68	11.60	<0.001
Predicted attractiveness from the nose	0.35	0.08	0.26	4.35	<0.001
Predicted attractiveness from the mouth	0.35	0.08	0.27	4.62	<0.001

**Table 8:** Correlations between the prediction error when predicting from the parts and the attractiveness of the parts in Study 2.

	Forehead	Eyes	Nose	Mouth
Prediction error from the forehead	0.46 ***	−0.52 ***	−0.32 **	−0.29 **
Prediction error from the eyes	0.08	0.70 ***	−0.12	−0.32 **
Prediction error from the nose	−0.18	−0.62 ***	0.20	−0.28 **
Prediction error from the mouth	0.04	−0.68 ***	−0.28 **	0.29 **

Note: ** *p* < 0.01; *** *p* < 0.001.

## Data Availability

The data generated and/or analyzed in this study are available from the corresponding author upon reasonable request.
